# Comprehensive Profiling of Blood Coagulation and Fibrinolysis Marker Reveals Elevated Plasmin-Antiplasmin Complexes in Parkinson’s Disease

**DOI:** 10.3390/biology10080716

**Published:** 2021-07-28

**Authors:** Amit Sharma, Jens Müller, Karin Schuetze, Verena Rolfes, Rosi Bissinger, Nathalia Rosero, Ashar Ahmad, Bernardo S Franklin, Berndt Zur, Holger Fröhlich, Florian Lang, Johannes Oldenburg, Bernd Pötzsch, Ullrich Wüllner

**Affiliations:** 1 Department of Neurology, University Hospital Bonn, 53127 Bonn, Germany; amit.sharma@ukbonn.de; 2 Institute of Experimental Hematology and Transfusion Medicine, University Hospital Bonn, 53127 Bonn, Germany; jens.mueller@ukbonn.de (J.M.); johannes.oldenburg@ukbonn.de (J.O.); bernd.poetzsch@ukb.uni-bonn.de (B.P.); 3 CellTool Gmbh, Am Neuland 1, 82347 Bernried, Germany; k.schuetze@celltool.de; 4 Institute of Innate Immunity, University Hospital Bonn, 53127 Bonn, Germany; rolfesv@gmail.com (V.R.); nathalia.rosero@uni-bonn.de (N.R.); franklin@uni-bonn.de (B.S.F.); 5 Department of Internal Medicine IV, Eberhard Karl University, 72076 Tuebingen, Germany; rosi.bissinger@uni-tuebingen.de; 6 Bonn-Aachen International Center for IT (B-IT), University Hospital Bonn, 53115 Bonn, Germany; ashar@bit.uni-bonn.de (A.A.); frohlich@bit.uni-bonn.de (H.F.); 7 Central Laboratory of the Rheinland Klinikum Neuss, 41464 Neuss, Germany; BZur@lukasneuss.de; 8 Department of Physiology, Eberhard Karls University, 72076 Tuebingen, Germany; florian.lang@uni-tuebingen.de; 9 German Center for Neurodegenerative Diseases (DZNE), 53127 Bonn, Germany

**Keywords:** Parkinson’s disease, blood coagulation, eryptosis, plasmin-antiplasmin complexes, homocysteine, haemoglobin, α-Synuclein, L-DOPA, platelets

## Abstract

**Simple Summary:**

A protein which was identified initially in nuclei and synapses of neurons and thus named alpha-synuclein (α-Syn) is significantly involved in Parkinson’s disease (PD). Mutations of the gene that codes for α-Syn cause familiar PD and α-Syn in sporadic PD has a tendency to form abnormal fibrils which are present in PD patients brains. α-Syn is also present in a variety of cells and biofluids, especially in red blood cells (erythrocytes), but also in plasma, saliva and platelets. This in turn raises the question whether presumably dysfunctional α-Syn affects the other enclosed constituents (haemoglobin, mediators of haemostasis, immunoglobulins and clotting factors). We thus searched for a potential impact in the aforementioned peripheral blood compartments in PD.

**Abstract:**

Parkinson’s disease (PD) is the second most common age-related neurodegenerative disease. Accumulating evidence demonstrates that alpha-synuclein (α-Syn), an apparently predominant neuronal protein, is a major contributor to PD pathology. As α-Syn is also highly abundant in blood, particularly in red blood cells (RBCs) and platelets, this in turn raises the question on the function of presumably dysfunctional α-Syn in “peripheral” cells and its putative effect on the other enclosed constituents. Herein, we detected the internal variance in erythrocytes of PD patients by Raman spectroscopy, but no measurable amount of erythrocytic behavioural change (eryptosis) or any haemoglobin variation was noticed. An elevated level of plasmin-antiplasmin complexes (PAP) was observed in the plasma of PD patients, indicating activation of the fibrinolytic system, but platelet activation after thrombin stimulation was not altered. Sex-specific patterns were noticed for blood coagulation factor XIII and factor XII activity in PD patients. Additionally, the alterations in homocysteine levels which have often been observed in PD patients were found to be independent from L-DOPA usage and PAP levels. Furthermore, a selective gene expression analysis identified subsets of genes related to different blood-associated compartments (RBCs, platelets, coagulation-fibrinolysis) also involved in PD-related pathways.

## 1. Introduction

Parkinson’s disease (PD) is a common neurodegenerative condition for which the exact molecular causes remain unknown, although several potentially relevant processes are being investigated [1,2,3]. Conceptualised as an ageing disorder of the nervous system, the disease process starts long before the appearance of typical motor symptoms and the overt PD phenotype develops slowly over time. No clear modifier or biochemical markers of progression have been identified, with the exception of a progressive decrease in dopaminergic cell function. A number of epidemiological studies have indicated an inverse association between the risk of developing malignancies and PD (except melanoma) [4,5].

Accumulating evidence demonstrates that α-Syn, an apparently predominant neuronal protein, is a major contributor to PD pathology. The structural conformation of this protein and various post-translational modifications play a significant role [6], as well as *SNCA* (the gene encoding α-syn) mutations and genomic multiplication, which cause familial forms of PD [7]. In addition to neurons, α-Syn is also present in a variety of cells and biofluids, including erythrocytes (red blood cells, RBCs) [8], plasma [9], saliva and platelets [10]. Of note, in a quantitative analysis RBCs, α-Syn (and DJ-1) range among the the 10 most abundand proteins [11]. In 2011, Selkoe and coworkers put forward the hypothesis of conformational changes of α-Syn, i.e increased monomers in PD in relation to a “healthy” helical tetramer confirmation, reminiscent of haemoblobin [12]. Although still under debate, this in turn raises the question on the function of presumably dysfunctional α-Syn in “peripheral” cells and its putative effect on the other enclosed constituents (erythrocytes: haemoglobin, methaemoglobin or pathophysiologically silent haemoglobin variants; platelets: several mediators of haemostasis/thrombosis; plasma: proteins, immunoglobulins, clotting factors/fibrinogens).

Considering the presence of altered α-Syn protein in PD patients, herein, we searched for its potential impact in the peripheral blood and found changes in PD subgroups based on Hcy and PAP levels. In addition, certain markers of coagulation physiology showed sex differences in PD. These parameters may point to yet unappreciated features of PD and warrant further investigation.

## 2. Material and Methods

### 2.1. Experimental Procedures

We examined the blood coagulation profile from clinically well-defined PD patients (males (*n* = 67), females (*n* = 28); age = 69 ± 4 years) and age-matched healthy controls (males (*n* = 35), females (*n* = 16); age = 68 ± 8 years). Hoehn and Yahr (HY) and the MMSE, respectively, were used to determine the level of disease severity (PD H&Y: 3.0 ± 0.7, range 2–4, median 3). All blood samples (10 mL) were obtained using routine venipuncture of a cubital vein and care was taken to avoid prolonged venous stasis in order to avoid activation of coagulation during sampling. The blood samples were processed within 1 h of collection. For preparation of plasma, filled blood tubes were centrifuged within 4 h at 2500× *g* for 15 min, and plasma samples were directly used or stored at <–40 °C until assayed. For determination of the activity of the plasmatic coagulation (co) factors, prothrombin time- (PT; factors II, V, VII and X) or activated partial thromboplastin time (aPTT; factors VIII, IX, XI and XII)-based clotting time assays were applied. In brief, patient plasma samples were diluted in the respective factor-deficient plasma (Siemens Healthcare Diagnostics, Marburg, Germany) and the mixtures were introduced to either the PT- or the aPTT-based assay, where activity levels were provided in percent of normal as determined by corresponding standard curves. Plasma levels of factor XIII activity, Fibrinogen, D-Dimer and von-Willebrand-Factor (vWF) activity were measured using the Berichrom FXIII, Multifibren^®^ U, Innovance D-Dimer and Innovance VWF Ac Testkit, respectively (all Siemens Healthcare Diagnostics). All aforementioned analyses were performed using a BCS XP coagulation analyser (Siemens Healthcare Diagnostics). Homocysteine plasma levels were measured on the Dimension Xpand platform (Siemens Healthcare Diagnostics) using the Homocysteine Enzymatic Assay (Siemens Dimension, Diazyme Europe, Dresden, Germany). Plasma levels of PAP-complexes, TAT-complexes, and t-PA were determined using the following ELISA tests: TECHNOZYM PAP, Complex and t-PA Ag EDTA ELISA Kits (Technoclone, Vienna, Austria) and the Enzygnost TAT micro-assay (Siemens Healthcare Diagnostics). The provided respective reference (normal) ranges (95% reference intervals) for each of the applied haemostasis assays were determined by analysing up to 100 plasma samples from healthy blood donors (M/F). For testing haemoglobin variants, 13 patients [males (*n* = 12), female (*n* = 1)] with PD were selected and the haemoglobin variants (HbA1a, HbA1b, HBF, HBS, HBC, LA1C, A1C, P3, A0, A2) were checked with HPLC to detect variations (Biorad, Munich, Germany). Methaemoglobin concentration (normal range −0.0–1.5%) was measured with a RAPIDPoint-500 analyser (Siemens Healthcare Diagnostics). After an interim analysis of these 13 patients, we stopped the experiments as no difference had become apparent. For statistical calculation, the significance level was set at *p* < 0.05 and indicated as * *p* < 0.05, ** *p* < 0.01, *** *p* < 0.001, **** *p* < 0.0001.

### 2.2. Raman Microscopy

Raman measurements were performed using an inverted Raman microscope (BioRam^®^- Raman laser trapping microscope, CellTool, Tutzing, Germany). Raman excitation was achieved by a 785 nm wavelength diode laser with 80 mw power (TOPTICA Photonics AG, Graefelfing, Germany). The scattered beam was collected by a 60x water immersion objective (1.1 NA, 0.2 WD) (Olympus, Hamburg, Germany) with a correction collar set to 0.17 mm. Detection was performed with a diffraction grating and a CCD detector (Andor, Belfast, UK). The blood cells were stabilised and captured during the measurements using the optical trapping force of the laser.

### 2.3. Raman Spectra Data Analysis

A collected Raman spectrum was first cropped to 600–1800 cm^−1^, as within this range most of the biological information can be found. The baseline was then corrected by an asymmetric least square fit, cosmic spikes were removed, and the spectra were smoothened with a median filter. Finally, the spectra were interpolated to continuous wave numbers and normalised using unit vector normalisation. Principal component analysis (PCA) was used for visualising the datasets. PCA score plots were used to find clusters among the data and PCA loadings enabled to find responsible wave number areas that showed the significant spectral variation between the analysed groups.

### 2.4. FACS Analysis of Annexin-V-Binding on Erythrocyte Surface

For determination of annexin-V-binding on the erythrocyte surface, we used blood samples from PD patients (*n* = 16) and age-matched healthy controls (*n* = 10). Briefly, 2 µL of freshly drawn blood were mixed in 500 µL Ringer solution containing 5 mM CaCl_2_, subsequently stained with Annexin-V-FITC (1:200 dilution; ImmunoTools, Friesoythe, Germany) at 37 °C for 15 min under protection from light. The annexin V abundance at the erythrocyte surface was subsequently determined using a FACS Calibur (BD, Heidelberg, Germany). Annexin-V-binding was measured with an excitation wavelength of 488 nm and an emission wavelength of 530 nm. A marker (M1) was placed to set an arbitrary threshold between annexin-V-binding cells and control cells. The same threshold was used for all erythrocytes. In total, 50,000 cells per sample were counted.

### 2.5. Platelet Isolation from Human Blood

Venous blood from Parkinson patients (*n* = 9) or age-matched healthy controls (*n* = 12) was drawn into S-Monovette^®^9NC collection tubes. The blood was centrifuged for 15 min at 330× *g* without brake to obtain platelet-rich plasma (PRP). All following centrifugation steps were performed without brake and in the presence of 200 nM PGE1 to inhibit (additional) platelet activation. PRP was transferred to a new tube and diluted 1:1 with phosphate-buffered saline (PBS) to reduce leukocyte contamination and centrifuged for 10 min at 240× *g*. Platelets were pelleted by centrifugation at 430× *g* for 15 min and washed once with PBS. Total platelets were counted using a haemocytometer and resuspended in RPMI medium to yield a concentration of 1 × 10^8^ cells/mL. Purified platelets were assessed by flow cytometry using CD45 (leukocyte) and CD41 (platelet) markers.

### 2.6. Assessment of the Activity of Isolated Platelets by Flow Cytometry

Samples of isolated platelets from Parkinson patients and healthy controls were analysed for purity, platelet pre-activation and platelet viability. Briefly, the platelets were activated with 1 U/mL thrombin for 30 min at 37 °C before the cells were blocked with 1:10 human Fc blocking reagent for 10 min at room temperature (RT). The samples were stained with fluorochrome-conjugated monoclonal antibodies against CD41/CD41, CD62P and CD45, as indicated, for 30 min in the dark. Cells were washed and resuspended in flow cytometry buffer (1% FBS in PBS) for analysis. Compensation beads (OneComp eBeads) and isotype controls were prepared in the same way. Flow cytometry was performed with a MacsQuant^®^ Analyser10 (Miltenyi Biotech, Bergisch Gladbach, Germany) and analysed using the FlowJo software (Tree Star). The applied gating strategy was based on doublet discrimination and isotype-matched control antibodies.

### 2.7. Gene Expression Analysis

Two publicly available PD-related gene expression datasets (GSE6613, GSE72267) based primarily on the whole-blood transcriptome were obtained from Gene Expression Omnibus [11,12]. The first dataset, GSE6613, was log-transformed, and one sample (GSM153508) was removed after quality control. This dataset contains 50 PD patients and 21 controls. The dataset GSE72267 contained already log-transformed values, and quality control once again led to the exclusion of one sample (GSM1859113). It contained 40 PD patients and 18 control subjects. The data were converted from Array Identifiers (AffyIDs) to Gene Symbols (HGNC) by averaging over the (log-) expression of all AffyIDs annotated to a single Gene Symbol. The annotation was built using ‘BioMart’. Limma analysis was then performed to estimate gene expression log-fold changes between PD and control subjects in both datasets [13]. We specifically used a set of 643 selective genes which—according to Gene Ontology annotation—were predicted to be related to RBC and haemoglobin function (oxygen transport, erythrocytic proteins), platelet activation-aggregation and blood coagulation-fibrinolysis pathways. Using the function ‘kegga’ from the R-package ‘limma’, we first conducted a gene set over-representation analysis for the PAP genes with respect to all available KEGG pathways [14]. This function computes one-sided hypergeometric tests. We then only considered those KEGG pathways which were statistically over-represented within these genes at a false discovery rate (FDR) threshold of 1%. We next performed Gene Set Enrichment Analysis (GSEA) to estimate the dysregulation of these selected pathways in both datasets. GSEA looks at a ranked list of genes and estimates how far genes annotated to a pathway of interest are statistically enriched at the beginning of the list. A high enrichment score along with the corresponding adjusted *p*-value (FDR) highlights the strong significance for that gene set. Our analysis resulted in 13 enriched KEGG pathways each for GSE6613 and GSE72267 at an FDR cut-off of 15%. In total, there were 7 common KEGG pathways between the 2 datasets, which contained 103 selective genes. The list of the common genes present in those pathways is presented in the Supplementary Materials of this paper.

## 3. Results and Discussion

Considering the presence of dysfunctional α-Syn in erythrocytes, we first focused on the enclosed/neighbouring haemoglobin and quantified the clinically silent and rare haemoglobin variants: HbA1a, HbA1b, HBF, HBS, HBC, LA1C, A1C, P3, A0 and A2 in PD patients, which did not reveal any abnormal peaks in the respective chromatograms (Supplementary Figure S1A,B). Moreover, we checked the concentration of methaemoglobin (FMetHb), but found no changes in FMetHb levels in the blood of PD patients compared to healthy controls (data not shown). Subsequently, we checked for any possible change in erythrocyte behaviour or eryptosis by examining cell membrane rearrangement (i.e., annexin-V-binding on the surface of erythrocytes), but found no significant differences (healthy volunteers: 0.56% ± 0.11%, PD patients: 0.48% ± 0.05%) (Supplementary Figure S1C), suggesting no signs of eryptosis in PD.

**Figure 1:**
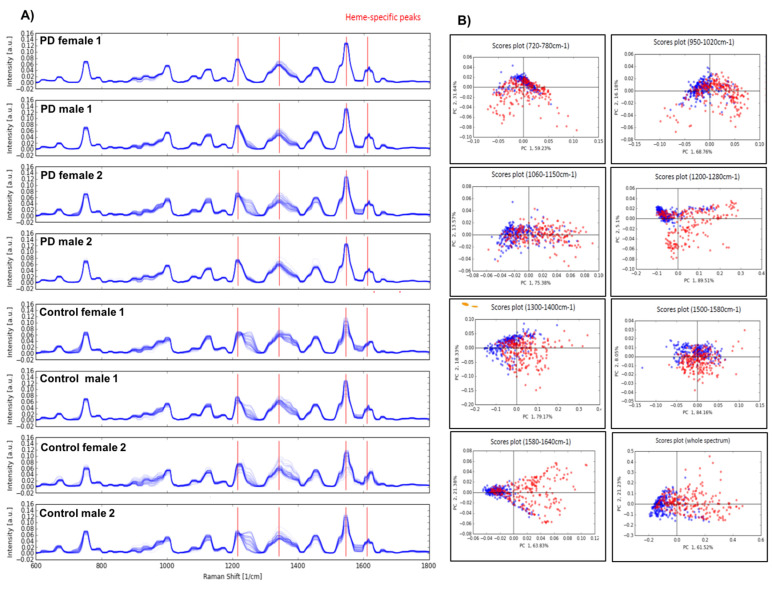
Raman spectroscopy of erythrocytes in PD. (**A**) Raman spectroscopy on the blood samples showed overall similar peaks constituting the spectrum, however internal variance in PD samples can be seen, which might be due to the disease-specific heterogeneity of single red blood cells. (**B**) Principal component analysis (PCA) clearly demonstrated that the Raman signal intensity at 1260 and 1545 cm^−1^ had the biggest correlation to the patient’s disease status.

To determine any possible intrinsic variability among the erythrocytes of PD patients, we performed Raman spectroscopy and found that the overall peaks constituting the spectrum were very similar in PD and controls. However, PD samples harboured considerable internal variance, which might be due to specific heterogeneity of single RBCs (Figure 1A, Supplementary Figure S2). Principal components analysis (PCA) suggested that the Raman signal intensity at 1260 and 1545 cm^−1^ might have a correlation with the proband’s disease status (Figure 1B). However, the initial analysis of the obtained spectroscopic changes in PD did not provide sufficient insights which would assist in establishing a more conclusive diagnostic biomarker. Therefore, we can conclude that α-Syn appears to have no effect on the neighbouring haemoglobin variants within the erythrocytes and it does not induce any altered erythrocyte behaviour in PD patients.

**Figure 2:**
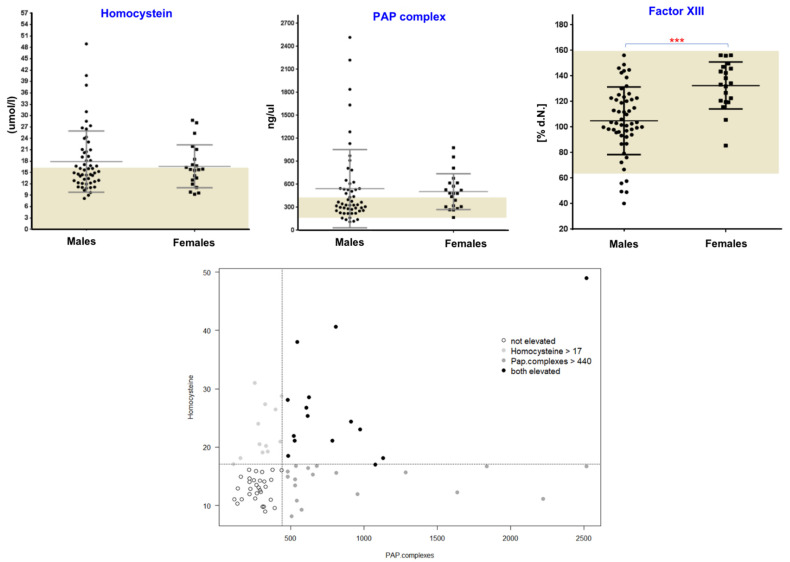
Evaluation of plasma homocysteine, PAP and factor XIII levels. Plots (upper section) showing values for PAP , homocysteine and factor XIII levels in PD patients. Dark bars illustrate the minimum to maximum range of levels for these markers in healthy populations. Three groups of patients with altered PAP or homocysteine levels are shown in the lower section. *p*-values < 0.05 were considered significant differences and are marked. (*** *p* < 0.001)

As mentioned above, in addition to red blood cells, other important blood compartments, such as plasma and platelets, also contain α-Syn, so minor or moderate changes directly in their function or associated factors can be expected in PD patients. We therefore investigated the plasma levels of various blood coagulation (co)factors and markers, such as factor II, factor V, factor VII, factor VIII, factor IX, factor X, factor XI, factor XII, factor XIII, fibrinogen, D-dimer, vWF, plasmin-Alpha2-antiplasmin (a2-AP) (PAP) complexes, thrombin-antithrombin III complexes (TAT) and tissue plasminogen activator (t-PA). In our analysis, we found a subgroup of patients with an increased PAP complex in both sexes (Figure 2). Given that the enhanced PAP complex can potentially impact fibrinolysis, we determined D-dimer levels (fibrinolysis marker) and observed changes only in a few PD patients; importantly, not all patients with elevated PAP levels showed D-dimer alterations (Supplementary Figure S4). A similar pattern was also observed in the case of the t-PA marker. This may partly be explained by the fact that the dynamic balance of several coagulation factors is the prerequisite for the initiation phase of fibrinolysis, and PD patients may lack this orchestration of all the factors. As enhanced activation of platelets can also contribute to the release of PAP, we evaluated platelet activation by thrombin and found that PD patients did not show any P-selectin (also known as CD62P) upregulation prior to thrombin treatment. Furthermore, after being activated by thrombin, platelets from both PD and healthy donors upregulated P-selectin to a similar degree, suggesting that platelet activation status is not the major cause of the increased PAP release observed (Supplementary Figure S3). In addition to P-selectin expression, we also assessed the purity of our samples by staining for the leukocyte marker CD45, which would indicate any platelet-leukocyte aggregates. The analysis showed extremely low CD45 levels (<1%), suggesting that platelet-leukocyte aggregates are present in very small amounts, if at all. Of note, DNA methylation levels of *SERPINF2* (the gene encoding for alpha-2-plasmin, one half-constituent of PAP complexes) have been previously linked to the onset of Alzheimer’s disease (AD) [15]. Therefore, we also investigated the promoter methylation status for *SERPINF2* in our previously performed Illumina 450 K analysis [16], but did not find any significant differences in PD patients when compared to healthy controls, suggesting that epigenetic-related changes, particularly in alpha-2-plasmin, may be restricted to AD, and are not a general phenomenon in neurodegenerative diseases (NDD).

Among the addressed coagulation factors, factor XIII (circulating transglutaminase), while within the normal range, showed significantly higher levels in female PD patients as compared to male subjects (*p* = 0.0003, Mann–Whitney test) (Figure 2). Previously, it had been suggested that tissue-specific transglutaminase (tTG) catalyses the formation of α-Syn crosslinks in PD [17], and oestrogens as a prothrombotic factor increase the levels of factor XIII with multiple other effects on the coagulation and fibrinolytic system [18,19]. Based on the results presented here, one could hypothesize that the contribution of circulating transglutaminase factor XIII might be non-specific, but rather represents a general sex-related phenomenon. Likewise, a weak but significant sex-specific difference was also observed in case of factor XII (Supplementary Figure S4). Recently, similar sex-specific differences were also discussed for other relevant blood-related markers in PD [20,21]. The investigation of several other coagulation markers showed the respective normal ranges with no significant differences between males and females or between healthy controls and PD patients (Supplementary Figure S4). However, a sex-specific trend was evident for factors V, IX, X and XI.

We also analysed plasma homocysteine (Hcy) levels, which are known to be elevated in PD, partly due to L-DOPA metabolism [22,23]. While we found increased Hcy levels in several PD patients, some PD patients were within the normal Hcy range (Figure 2). In our analysis, there was no clear-cut relationship between the L-DOPA usage (data not shown) and elevated Hcy and PAP levels in this cohort, which point towards additional mechanisms such as Vitamin B status or L-DOPA-independent pathways which might be unique for PD. Overall, PD patients showed three subgroups based on Hcy and PAP levels: high Hcy and high PAP (group 1), high Hcy and normal PAP (group 2) and normal Hcy and high PAP (group 3) (Figure 2). Despite the fact that we observed three subgroups based on Hcy and PAP levels, there was no association with FXIII, as PD patients with different FXIII levels were randomly distributed across all these subgroups.

In an attempt to substantiate our findings described above, we took advantage of two publicly available gene expression studies, performed in the blood samples of PD, and checked the expression status of 643 selective genes, which are predicted to be related to RBC and haemoglobin function (oxygen transport, erythrocytic proteins), platelet activation-aggregation and blood coagulation-fibrinolysis pathways by the Gene Ontology database (GO (http://www.geneontology.org, accessed on 20 February 2019). In our analysis, we could identify 7 KEGG (Kyoto Encyclopaedia of Genes and Genomes) pathways that were commonly upregulated in both studies (Supplementary Figure S5, Supplementary Table S1). These pathways included focal adhesion, which has previously been identified in a consensus from several GWAS studies of PD patients, immune system-related pathways (natural killer cell-mediated cytotoxicity, leukocyte trans-endothelial migration, chemokine signalling), osteoclast differentiation and Systemic Lupus Erythematosus (SLE). This is consistent with recent studies associating PD with neuroinflammation [24], Systemic Lupus Erythematosus [25] and higher risk of osteoporosis [26]. Altogether, our selective gene expression analysis also demonstrated that a subset of genes that play an active role in RBCs, haemoglobin, platelet activation-aggregation and blood coagulation-fibrinolysis are altered and contribute significantly in PD-associated key biological pathways.

Taken together, as blood is easily obtained compared to neuronal tissues, we aimed to phenotypically identify a presumed dysfunction of α-Syn in peripheral blood compartments in PD patients. We classified PD patients into subgroups on the basis of Hcy and PAP levels and observed sex differences in certain coagulation markers. Hopefully, the proposed parameters will help clinics to identify more such patients and to further investigate the factors driving these variations in subgroups of PD patients.

## 4. Conclusions

Starting from hypotheses about the presence of haemoglobin variants or eryptosis in RBCs of PD patients, our systematic investigations did not reveal a clear association. However, we observed an internal variance in RBCs of PD patients and found alterations in plasma levels of PAP complexes, which were not associated with L-DOPA usage or elevated plasma homocysteine levels in PD. Whether these changes could lead to a relevant biomarker must be evaluated in larger patient cohorts.

## Data Availability

The data that support the findings of this study are available from the corresponding authors upon reasonable request.

## Supplementary Materials

The following are available online at https://www.mdpi.com/article/10.3390/biology10080716/s1, Figure S1: Chromatogram to detect Haemoglobin variants and percentage of annexin-V-binding erythrocytes (Eryptosis measurement). Figure S2: Normalised Raman spectra (Raman Shift) of erythrocytes in PD and healthy controls. Figure S3: Platelets activation in PD patients and healthy controls. Figure S4: Blood coagulation markers showing normal levels in PD patients. Figure S5: Gene expression analysis and pathway enrichment. Table S1: List of 103 common genes identified in KEGG pathways.
