# Supplementary material for: Comprehensive Profiling of Blood Coagulation and Fibrinolysis Marker Reveals Elevated Plasmin-Antiplasmin Complexes in Parkinson’s Disease
Source: Biology (Basel). 2021 Jul 28;10(8):716. doi: 10.3390/biology10080716 (PMC8389253; doi:10.3390/biology10080716)
Supplement: Supplementary file 1 [file biology-10-00716-s001.zip › biology-1243740-supplementary/Supplementary/Supplementary Table S1.pdf]

Supplementary Table S1

Entrez ID	Gene Name	Entrez ID	Gene Name
60	ACTB	5026	P2RX5
71	ACTG1	9127	P2RX6
87	ACTN1	5027	P2RX7
135	ADORA2A	5028	P2RY1
150	ADRA2A	5154	PDGFA
151	ADRA2B	5155	PDGFB
152	ADRA2C	56034	PDGFC
207	AKT1	80310	PDGFD
408	ARRB1	5156	PDGFRA
836	CASP3	5159	PDGFRB
958	CD40	5170	PDPK1
959	CD40LG	5175	PECAM1
4267	CD99	5290	PIK3CA
998	CDC42	5291	PIK3CB
1535	CYBA	5294	PIK3CG
1536	CYBB	5295	PIK3R1
2147	F2	23533	PIK3R5
2149	F2R	5336	PLCG2
2150	F2RL1	5340	PLG
2151	F2RL2	5566	PRKACA
2335	FN1	5578	PRKCA
2534	FYN	5567	PRKACB
3020	H3F3A	5568	PRKACG
8334	HIST1H2AC	5579	PRKCB
85236	HIST1H2BK	5580	PRKCD
8342	HIST1H2BM	5582	PRKCG
8350	HIST1H3A	5724	PTAFR
8357	HIST1H3H	5728	PTEN
8337	HIST2H2AA3	5739	PTGIR
8349	HIST2H2BE	5781	PTPN11
3456	IFNB1	5879	RAC1
3635	INPP5D	5880	RAC2
3673	ITGA2	5894	RAF1
3674	ITGA2B	2889	RAPGEF1
3688	ITGB1	387	RHOA
3690	ITGB3	6688	SPI1
3815	KIT	6772	STAT1
3932	LCK	6850	SYK
3937	LCP2	6915	TBXA2R
4067	LYN	7006	TEC
5594	MAPK1	7040	TGFB1
1432	MAPK14	7067	THRA
5595	MAPK3	706	TSPO
4149	MAX	7294	TXK
4609	MYC	7409	VAV1
10627	MYL12A	7410	VAV2
10398	MYL9	10451	VAV3
5023	P2RX1	7414	VCL
22953	P2RX2	7422	VEGFA
5024	P2RX3	7450	VWF
5025	P2RX4	7454	WAS
