# Supplementary figures and images for: Comprehensive Profiling of Blood Coagulation and Fibrinolysis Marker Reveals Elevated Plasmin-Antiplasmin Complexes in Parkinson’s Disease
Source: Biology (Basel). 2021 Jul 28;10(8):716. doi: 10.3390/biology10080716 (PMC8389253; doi:10.3390/biology10080716)

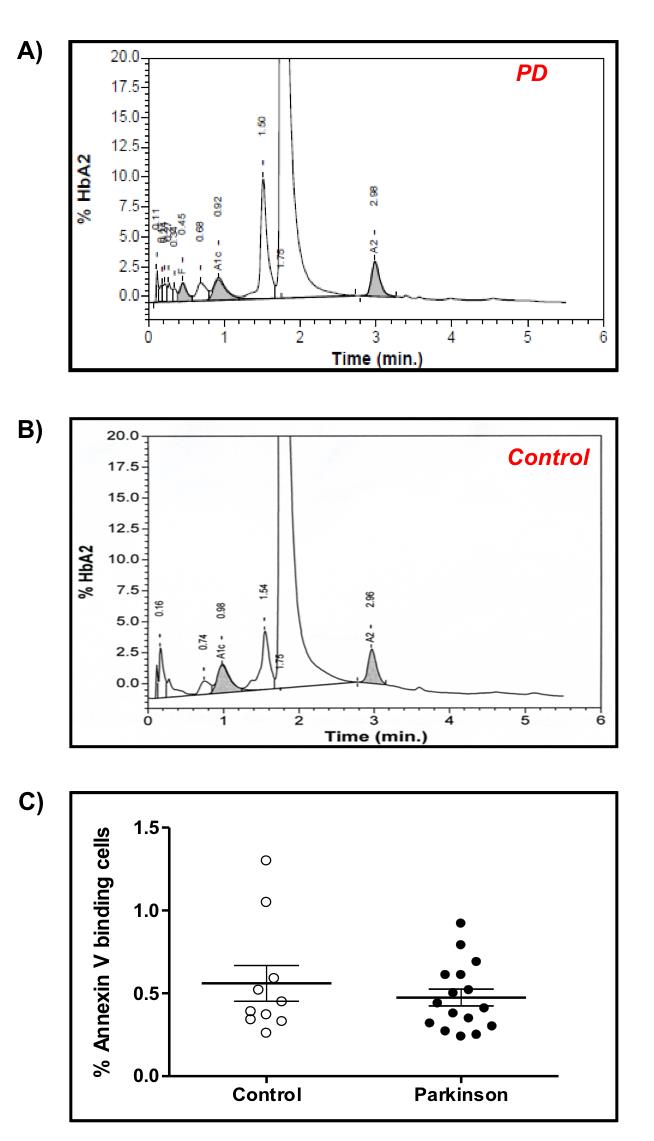

Supplement: Supplementary file 1 [file biology-10-00716-s001.zip › biology-1243740-supplementary/Supplementary/Supplementary figure S1.jpg]

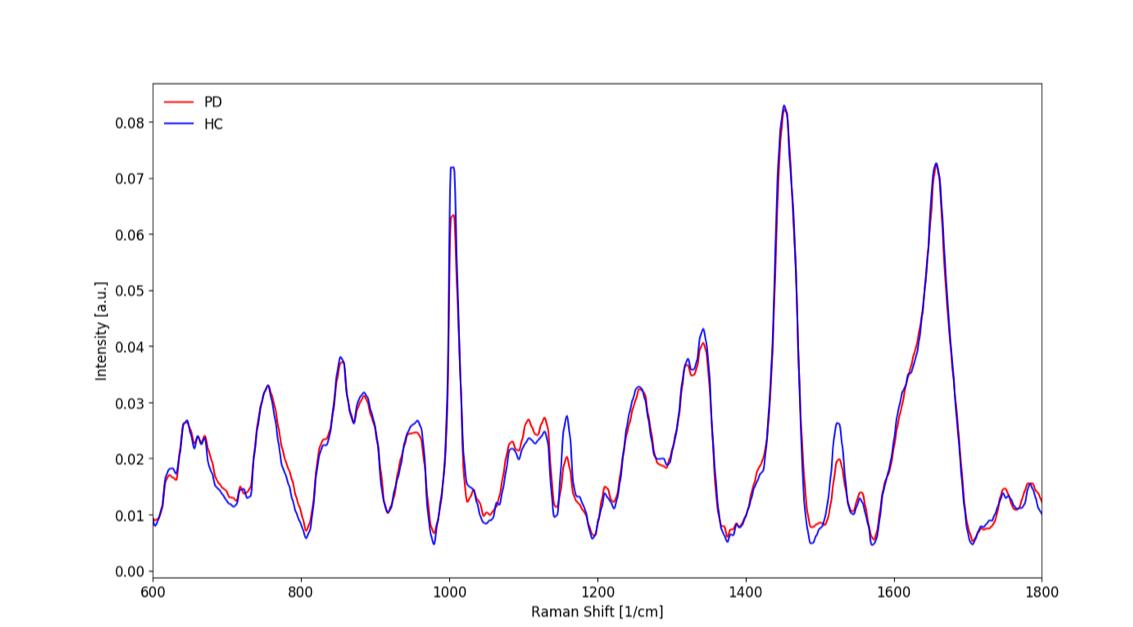

Supplement: Supplementary file 1 [file biology-10-00716-s001.zip › biology-1243740-supplementary/Supplementary/Supplementary figure S2.jpg]

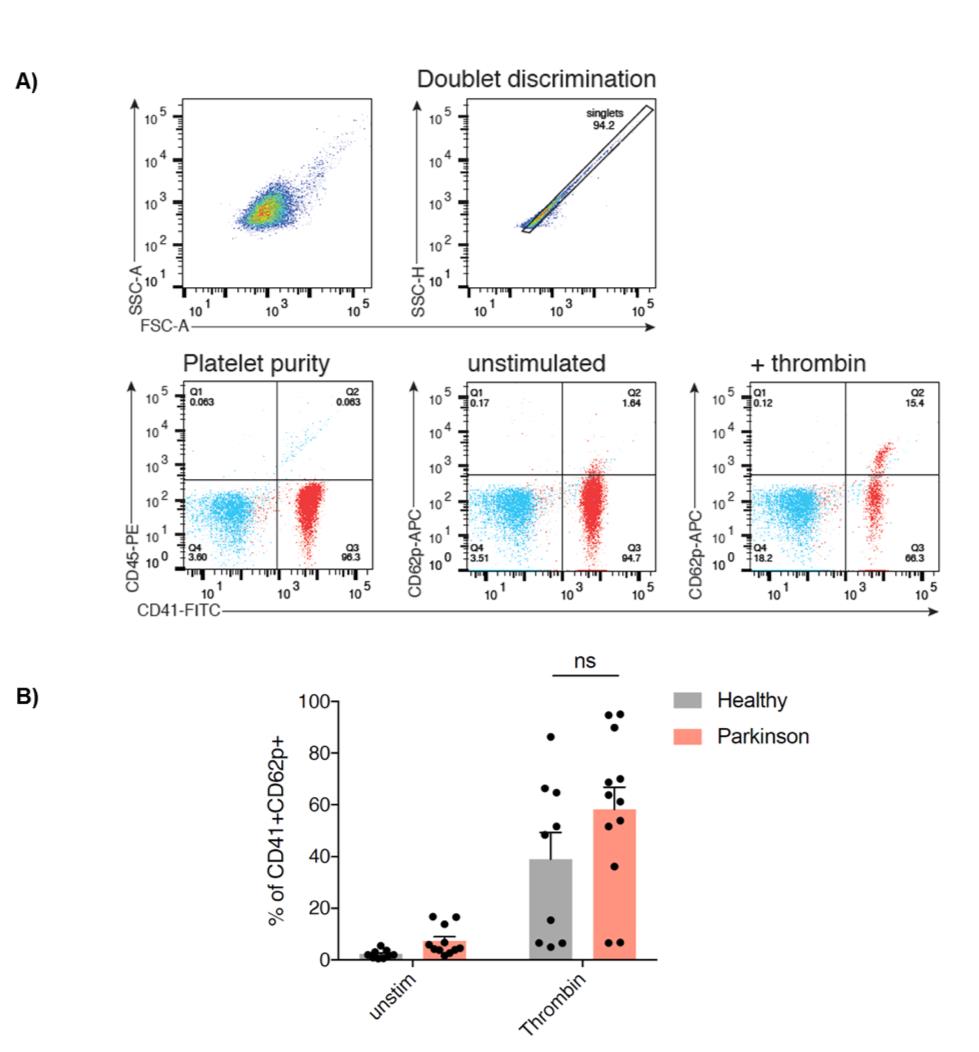

Supplement: Supplementary file 1 [file biology-10-00716-s001.zip › biology-1243740-supplementary/Supplementary/Supplementary figure S3.jpg]

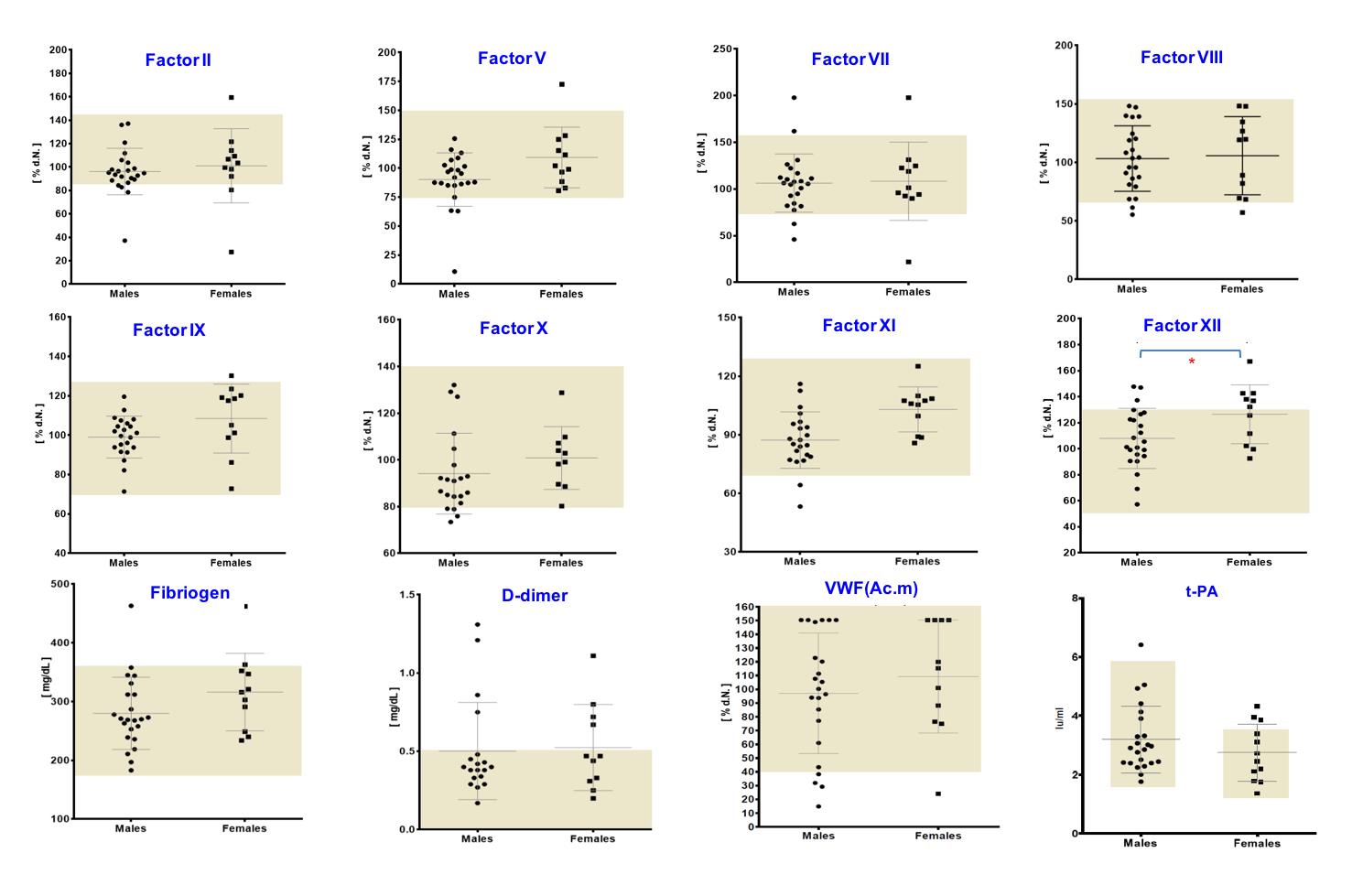

Supplement: Supplementary file 1 [file biology-10-00716-s001.zip › biology-1243740-supplementary/Supplementary/Supplementary figure S4.jpg]

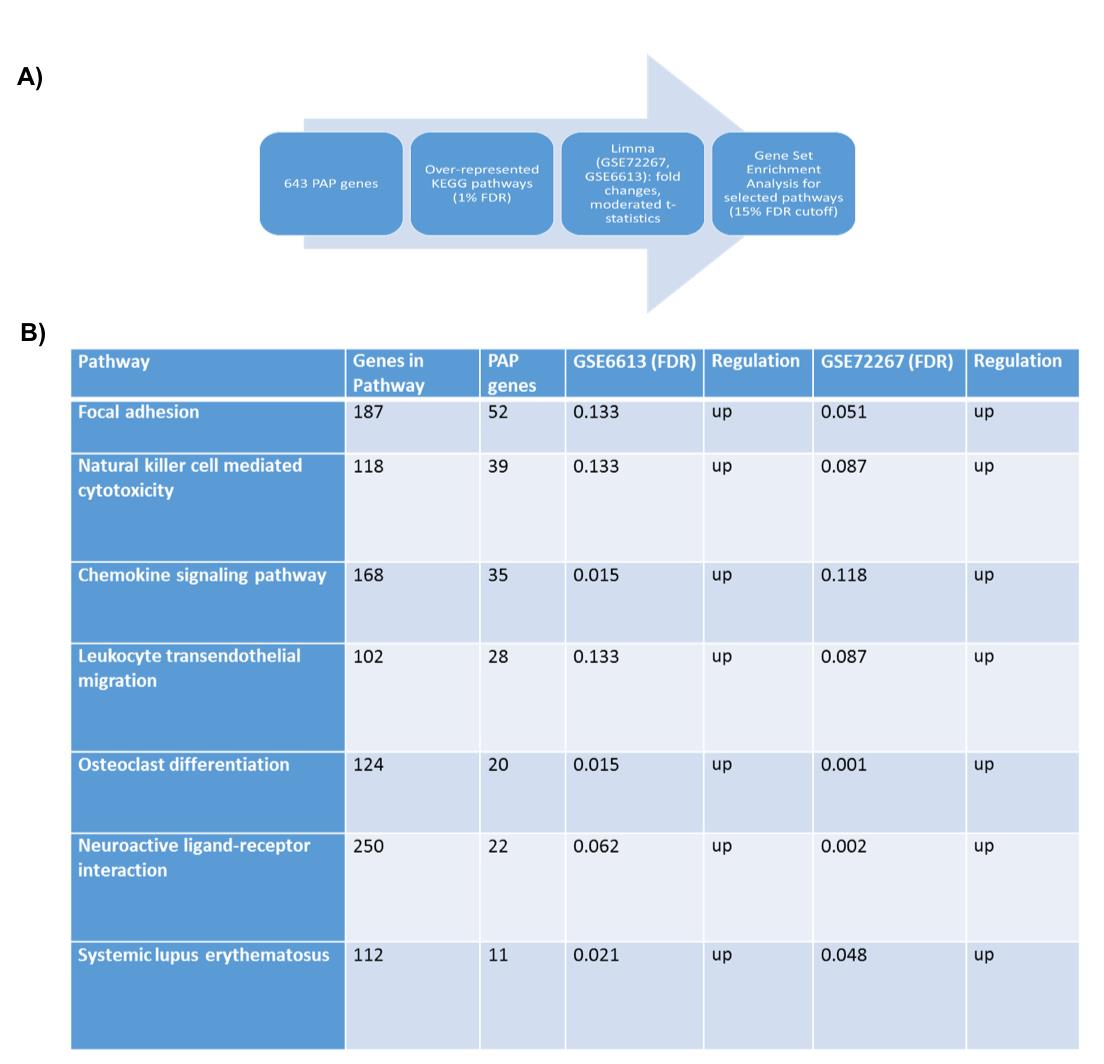

Supplement: Supplementary file 1 [file biology-10-00716-s001.zip › biology-1243740-supplementary/Supplementary/Supplementary figure S5.jpg]
